# New Aspects of Lipotoxicity in Nonalcoholic Steatohepatitis

**DOI:** 10.3390/ijms19072034

**Published:** 2018-07-13

**Authors:** Nahum Mendez-Sanchez, Vania Cesar Cruz-Ramon, Oscar Lenin Ramirez-Perez, Jessica P. Hwang, Beatriz Barranco-Fragoso, Jaqueline Cordova-Gallardo

**Affiliations:** 1 Liver Research Unit, Medica Sur Clinic & Foundation, 14050 Mexico City, Mexico; vaniacesar_ram55@hotmail.com (V.C.C.-R.); oscar.tuzos@hotmail.com (O.L.R.-P.); 2 Department of General Internal Medicine, The University of Texas MD Anderson Cancer Center, Houston, TX 77030, USA; jphwang@mdanderson.org; 3 Department of Gastroenterology, National Medical Center “20 Noviembre”, 03229 Mexico City, Mexico; betbarranco@yahoo.com.mx; 4 Obesity Clinic, General Hospital “Dr. Manuel Gea González”, 14080 Mexico City, Mexico; jacquiemex2@yahoo.com.mx

**Keywords:** nonalcoholic steatohepatitis, liver fibrosis, hepatic lipotoxicity

## Abstract

NASH is becoming increasingly common worldwide because of the growing global prevalence of obesity and consequently NAFLD. Unfortunately, the mechanism of progression of NAFLD to NASH and then cirrhosis is not completely understood. Several factors, including insulin resistance, inflammation, oxidative stress, lipotoxicity, and bile acid (BA) toxicity, have been reported to be associated with NASH progression. The release of fatty acids from dysfunctional and insulin-resistant adipocytes results in lipotoxicity, which is caused by the ectopic accumulation of triglyceride-derived toxic metabolites and the subsequent activation of inflammatory pathways, cellular dysfunction, and lipoapoptosis. Adipose tissue (AT), especially visceral AT, comprises multiple cell populations that produce adipokines and insulin-like growth factor, plus macrophages and other immune cells that stimulate the development of lipotoxic liver disease. These biomolecules have been recently linked with many digestive diseases and gastrointestinal malignancies such as hepatocellular carcinoma. This made us question what role lipotoxicity has in the natural history of liver fibrosis. Therefore, this review focuses on the close relationship between AT and NASH. A good comprehension of the pathways that are related to dysregulated AT, metabolic dysfunction, and hepatic lipotoxicity will result in the development of prevention strategies and promising therapeutics for patients with NASH.

## 1. Introduction

The incidence of obesity is reaching epidemic proportions. Consequently, the global incidence of secondary diseases such as metabolic syndrome (MetS), type 2 diabetes mellitus (T2DM), nonalcoholic fatty liver disease (NAFLD), and nonalcoholic steatohepatitis (NASH) has become very high [1]. NAFLD comprises a wide spectrum of conditions related to over accumulation of lipids in the liver, which range from steatosis to NASH [2]. NASH is characterized by the accumulation of fat in the liver in parallel with hepatocyte damage, inflammation, and different degrees of scarring or fibrosis, and has a high risk of progressing to cirrhosis and hepatocellular carcinoma [3].

Several factors, including lipotoxicity, insulin resistance, inflammation, oxidative stress, and bile acid (BA) toxicity, have been associated with NASH progression [4]. Nevertheless, the molecular mechanisms that promote NASH progression remain poorly understood. This lack of knowledge limits the development of preventatives and therapeutics for this disease. Previous studies have proposed several models to explain the molecular events in NAFLD-induced liver injury [5,6].

Lipotoxicity is the harmful effects of lipid accumulation in non-adipose tissue. It is well known that lipotoxicity is related to insulin resistance, hepatic steatosis, cardiomyopathy, nephropathy, and endothelial dysfunction [7]. Nowadays, the development and progression of NASH is mainly viewed as the consequence of liver lipotoxicity. Hepatic lipotoxicity occurs when the liver capacity to utilize, store, and export free fatty acids (FFAs) as triglycerides (TGs) is overcome by FFAs flux from the periphery or hepatic *de novo* lipogenesis [8]. Recent data show that storage of TGs in liver cells is not the leading determinant of lipotoxicity, and that FFAs act as harmful agents in hepatocytes [9]. This review discusses the new approaches of molecular mechanisms of lipid-induced hepatocellular damage.

## 2. The Light and Dark Sides of Adipose Tissue

Adipose tissue (AT) is a connective tissue that is comprised mostly of fat storage cells, but also contains water, minerals, and proteins [10]. The average volume of AT in adult human life is approximately 24 L whereas the liver has a volume of 1.5–1.8 L [11]. Obesity increases the volume of AT, which may surpass 80 L in the morbidly obese [10].

AT is classified as subcutaneous or intra-abdominal depending on its anatomical position [12]. Subcutaneous AT is located under the skin and stores approximately 80% of total body fat. Intra-abdominal AT includes visceral AT, which is located around the digestive organs, and retroperitoneal AT, which together account for 20% of total body fat [13]. There are also two types of AT: white AT (WAT) and brown AT (BAT). The main function of WAT is to store energy, while BAT is adapted to generate body heat. BAT is practically nonexistent in adults but is very common in fetuses and newborns [14]. WAT is a secretory organ that both sends and receives signals to modulate energy expenditure, sensitivity to insulin, appetite, endocrine and reproductive functions, and inflammatory and immunological processes [15]. A feature of subcutaneous AT is that it is less sensitive to the action of insulin than is visceral AT [16,17]. Therefore, visceral adiposity is more often related to NAFLD and MetS. The lipolytic effect of catecholamines is stronger in visceral adipocytes and the antilipolytic effect of insulin is weaker, which leads to greater mobilization of FFAs for lipolysis in intra-abdominal AT than in subcutaneous AT [18]. Some studies have observed that visceral AT has a higher impact on systemic inflammation and an adverse metabolic profile compared with other AT because visceral AT is more inclined to become inflamed in obese individuals [15,18].

**Figure 1:**
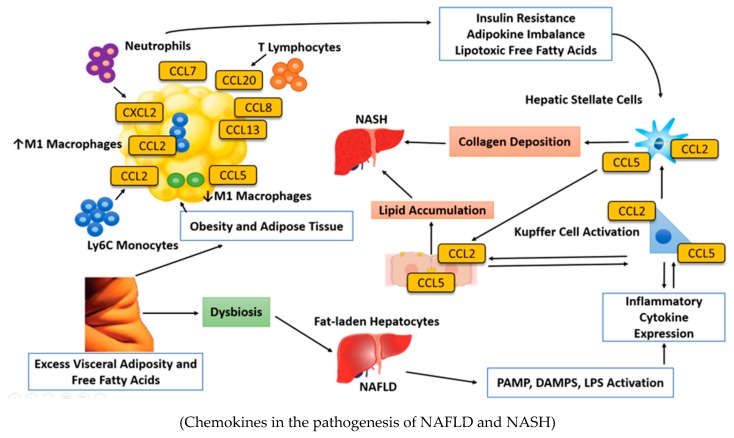
The pathogenesis of nonalcoholic steatohepatitis (NASH) is not completely understood. It seems the mechanism leading to NASH is multifactorial. Obesity is a strong contributing factor to nonalcoholic fatty liver disease (NAFLD) progression due to liver steatosis is related to lipotoxicity and cellular stress, which result in hepatocyte injury. Evidence has shown a strong relationship between obesity and gut microbiota configuration in humans [21,22]. Obesity has been related to modify the gut microbiota and gut microbiota has demonstrated a modifier role of metabolic and end organ complications of obesity such as NAFLD/NASH. The imbalance of gut microbiota (dysbiosis) leads to increased pathogen-associated molecular patterns (PAMPs). Moreover, dysbiosis is related to an increased exposure to bacterial products coming from the gut such as gram-negative-derived lipopolysaccharides (LPS). Molecular pattern molecules (DAMPs) and PAMPs act on receptors localized on hepatic cells leading to recruitment of neutrophils, macrophages, and other components of the innate immune response. Kupffer cells are sensors of liver tissue damage and they become activated through PAMPs, by increased levels of LPS and by DAMPs. The activated Kupffer cells release proinflammatory cytokines and chemotactic factors such as chemokine C-C motif ligand (CCL). Consequently, hepatic stellate cells are activated. Finally, this process causes an immoderate production of extracellular matrix leading to progressive fibrosis.

The adipocyte is the functional unit of AT, but comprises only 20–40% of the cellular content of AT [17]. It is important to note that AT includes other cell types such as the stroma vascular fraction, which includes blood cells, endothelial cells, pericytes, and adipocyte precursor cells, among others [18]. The main role of adipocytes is lipid uptake and storage. However, AT is no longer considered to be a tissue that only stores fat. Recent evidence has shown that AT is a major endocrine organ because it is responsible for the synthesis and secretion of many hormones and inflammatory cytokines (adipokines) [19]. Concentrations of these biological substances are higher in visceral AT compared with subcutaneous AT [20]. This is highly significant because obese individuals have been suggested to be in a state of low-grade inflammation, which dysregulates AT, causing an alteration of adipokine production and thereby linking obesity, insulin resistance, inflammation, and NAFLD [20] (Figure 1).

It has been recognized that among other processes, AT participates in the regulation of nutritional intake, immunity, angiogenesis, fibrinolysis, and control of sensitivity to insulin [20].

The correct functioning of adipocytes depends on many factors, such as adipocyte size, number, hormonal milieu, and the interplay between AT and other cell types [23]. Adipocytes derive from multipotent mesenchymal stem cells, which develop into adipoblasts and subsequently into preadipocytes. Preadipocytes can differentiate into mature adipocytes, thus enabling hyperplastic expansion of AT when increased storage is needed [24]. The mature adipocytes can elongate to adapt to increased storage needs, and in situations of overfeeding, become hypertrophic [25]. Thus, adipocyte number and morphology change in response to the energy balance by means of biochemical processes implicated in lipid uptake, esterification, lipolysis, and differentiation of preadipocytes [26].

Adipocyte maturation depends on upregulation of peroxisome proliferator-activated receptor gamma (PPAR-γ) together with other transcription factors including sterol regulatory-element binding protein (SREBP) 1c, cytosine–cytosine–adenosine–adenosine–thymidine (CCAAT)-enhancer-binding proteins, and bone morphogenetic protein [19]. These transcription factors are very important because they give adipocytes their plasticity and confer a significant ability to adapt to high-fat overfeeding through hypertrophy and hyperplasia. Accordingly, the AT is a protective tissue that stores and prevents immoderate exposure of other organs to FFAs [19]. To avoid lipotoxicity from chronic overnutrition, adipocytes first become hypertrophic then hyperplastic. It has been observed that hypertrophic adipocytes develop a pattern of gene expression that resembles that of macrophages and that they produce adipokines [26]. Consequently, protection from chronic overfeeding and from triglyceride accumulation in tissues such as the liver, muscle, and pancreatic β-cells requires an extraordinary adaptation by adipocytes that implicates activation of several inflammatory pathways but comes at the cost of AT insulin resistance [27].

## 3. Lipids: From Synthesis to Breakdown

The accumulation of fat depends on the balance between fat synthesis (lipogenesis) and fat breakdown (lipolysis) [28]. Lipogenesis can occur in AT or in the liver. Lipogenesis controls the synthesis of FFAs and is stimulated by a diet including high levels of carbohydrates conducive to high postprandial plasma TGs levels, whereas it is inhibited by polyunsaturated FFAs and by fasting [29]. Lipolysis occurs in AT and is responsible for producing energy from energy reserves (TGs), during which triacylglycerol molecules are hydrolyzed to FFAs and glycerol [30]. During fasting or prolonged hard exercise, the adipocyte’s TG droplet is degraded to supply FFAs to be used as an energy source by other tissues [31]. Hormone-sensitive lipase and monoacylglycerol lipase are responsible for catalyzing the hydrolysis of the TGs ester bonds [31]. Complete hydrolysis of TGs involves the breaking of three ester bonds to release FFAs and a glycerol moiety [32]. The hormone-sensitive lipase is responsible for facilitating hydrolysis of the esters at positions 1 and 3 of the triacylglycerol, while 2-monoacylglycerol lipase catalyzes hydrolysis of the remaining ester to produce a third FFA and glycerol. Insulin inhibits the hormone-sensitive lipase and the secretion of this hormone is favored by the presence of glucagon and epinephrine B [33]. Glycerol is transported out of adipocytes via an aquaporin type of transport molecule and must be shuttled back to the liver for use in oxidation or gluconeogenesis [34]. Outside the adipocyte, FFAs are instantly bound to albumin and carried in the bloodstream to the liver, muscle, and other tissues for oxidation. β-oxidation is a catabolic process in which the FFAs resulting from lipolysis are used as a source of energy [35]. Finally, the FFA molecules are converted into acetyl coenzyme A (CoA) molecules.

## 4. Lipotoxicity

Lipotoxicity is characterized by an increased concentration of toxic lipids and lipids derivatives [28]. Overnutrition with high-carbohydrate and high-fat diets is related to the development of one of two clinical–histopathological phenotypes of liver fatty accumulation: NAFLD or NASH [36]. Many types of lipid have been demonstrated to mediate liver lipotoxicity [28], including FFAs, TGs, free cholesterol (FC), lysophosphatidyl cholines (LPCs), ceramides, and BA. We describe the roles of these biological molecules below.

### 4.1. Free Fatty Acids

FFAs can be classified as saturated or unsaturated, depending on the bonds. They are unsaturated if they contain double bonds, while those without double bonds are called saturated [37]. It is well known the several roles of FFAs such as energetic substrates, components of membrane lipids, as ligands of some membrane receptors and as substrates for the synthesis of signaling molecules and complex lipids [37]. The most plentiful FFAs in the diet and in the steatotic liver are saturated palmitic acid (PA) and monounsaturated oleic acid (OA). PA is ingested as part of the diet or can be produced by lipogenesis from excess carbohydrate consumption. OA can be found in animal and vegetable fats and oils and is the most abundant FFAs in AT [28]. It has been observed in vitro models that OA or PA can induce steatosis separately as well as in a mixture [38]. Some studies have shown harmful effects of unsaturated FFAs on hepatocytes via induction of apoptosis [38,39]. Liver cells exposed to elevated levels of circulating FFAs raise their uptake to clear the FFAs from the blood [40]. Most of the FFAs that enter the liver originate from lipolysis of AT and TGs in the fasting state, and constitute 60% of liver FFAs in people with NAFLD [41]. It has been shown that hepatocytes exposed to PA and OA display increased expression of fatty acid translocase (also known as CD36) and fatty acid transport protein-2, leading to accumulation of diacylglycerol (DAG) or ceramides into the cells [39,42].

### 4.2. Triglycerides

TGs are the main component of dietary lipids and represent the main form of energy storage. TGs are simple lipids that are composed of a glycerol molecule associated with three FFAs [41]. Human in vivo studies have shown that excess accumulation of hepatic TGs is the main consequence of increased delivery of adipose-derived FFAs to the liver and *de novo* lipid synthesis in the liver [28,41]. On the other hand, liver steatosis is affected by lipid disposal via β-oxidation or export of very low density lipoproteins or directly by the increment of dietary lipids. High-carbohydrate diets contribute to liver steatosis by facilitating lipogenesis and lipid storage as TGs [43]. A protective role for TGs has also been demonstrated: it seems that TGs represent a defense system against the proapoptotic effects of high loads of FFAs in cells [44]. In this regard, Yamaguchi et al. demonstrated the protective role of TGs by showing that inhibition of DAG acetyltransferase 2, the final catalyst in hepatocyte TG synthesis, generated increased necroinflammation and increased peroxidation and oxidative stress [45].

### 4.3. Lysophosphatidyl Cholines

LPCs are plasma lipid constituents derived from phosphatidylcholine. LPCs have been reported to be involved in phagocyte chemotaxis and are released after activation of calcium-independent phospholipase A2 by caspase 3 when cells undergo apoptosis [46]. It has been observed that levels of LPCs are greater in the hepatic tissue or plasma of both human NASH patients and in animal models of NASH [47,48]. Moreover, LPCs seem to be a key mediator of FFAs cytotoxicity by causing an endoplasmic reticulum (ER) stress and inducing apoptotic pathways downstream of the activation of Jun terminal kinase (JNK) or glycogen synthase kinase 3 and the induction of the transcription factor CCAAT/enhancer-binding homologous protein [48,49]. These processes lead to an upregulation of prodeath proteins.

### 4.4. Ceramides

Ceramides are members of the sphingolipid family of lipids and components of the cell membranes. Ceramides are involved in several processes such as cell signaling, inhibition of insulin signaling, apoptosis, induction of oxidative stress and inflammation. High circulating levels of ceramides were observed in peripheral blood samples of obese patients with NASH [50]. Ceramides can be produced through three different pathways: *de novo* synthesis, a sphingomyelinase pathway, and a salvage pathway. *De novo* synthesis from palmitoyl CoA and serine subjected to oxidative stress through the action of serine palmitoyl CoA transferase, which is the rate-limiting enzyme [50,51]. Ceramides can be produced by the action of neutral sphingomyelinase (N-SMase)-mediated hydrolysis of cell membrane sphingomyelin. Inflammation upregulates activity of N-SMase [52]. Glutathione can be a negative regulator of N-SMase. Decreased levels of glutathione lead to the activation of N-SMase [52]. In the salvage pathway, ceramides are recycled by the ceramide synthase catalyzed reacylation of sphingosine generated from catabolism of complex sphingolipids [50]. Fumanisin B1 is an inhibitor of (dihydro) ceramide synthase that can inhibit both ceramide biosynthesis and the salvage pathways [50].

Increased levels of pro-inflammatory cytokines such as interleukin (IL)-1 and IL-6 are present in people with NASH and have a relationship with increased ceramide levels [51,52]. Ceramides are related to tumor necrosis factor alpha (TNFα) in the liver and promote the release of reactive oxygen species (ROS) by hepatic mitochondria, resulting in apoptosis, and worse, hepatic inflammation [53]. Increased liver ceramide levels have also been associated with the development of hepatic insulin resistance due to ceramides can attenuate insulin signaling in liver and skeletal muscle by reducing GLUT4 translocation and glucose uptake [54]. The function of ceramides in NAFLD pathogenesis and lipotoxicity is still not fully understood but seems to be cell-type-specific [55]. Hepatocyte apoptosis induced by saturated FFAs was found to be ceramide independent, whereas in pancreatic β-cells, ceramides are important mediators of cellular failure and apoptosis and may function as a second messenger in cross-talk between the ER and mitochondria [56].

### 4.5. Free Cholesterol and Bile Acids

Nowadays, it is known that the alteration of liver cholesterol homeostasis and hepatic FC accumulation are crucial to the pathogenesis of NAFLD/NASH.

In NAFLD, the accumulation of hepatic FC results from alterations in intracellular cholesterol transport and from unbalanced cellular cholesterol homeostasis characterized by activation of cholesterol biosynthetic pathways, increased cholesterol de-esterification and decreased cholesterol export and BA synthesis [57]. High intracellular FC leads to liver damage through the activation of Kupffer cells and hepatic stellate cells, which mediate inflammation and fibrosis [57]. In addition, the accumulation of FC in liver mitochondria induces mitochondrial dysfunction, which results in increasing generation of ROS and in activation of the unfolded protein response in the endoplasmic reticulum (ER), resulting in ER stress and hepatocyte apoptosis [57]. In the liver, the breakdown of cholesterol homeostasis contributes to its accumulation in hepatocytes and within organelles [57]. These events contribute to the maintenance of steatosis and promote ongoing hepatocyte death and liver injury.

SREBP-2 is the main regulatory enzyme of hydroxymethylglutaryl-CoA reductase, which is a key enzyme in cholesterol synthesis [58]. SREBP-2 levels are increased in NASH patients, correlating with high levels of FC and steroidogenic acute regulatory protein (a mitochondrial cholesterol transporter) [59]. In vitro models, it has been demonstrated that diets with high levels of FC cause accumulation of toxic hepatic oxysterols, which induce to mitochondrial dysfunction and liver damage [60]. In addition, FC accumulation in liver mitochondria led to apoptosis by a Toll-like receptor 4 (TLR4)-regulated JNK-1 pathway that activated the inflammatory mediator high mobility group box 1 protein. The relationship between cholesterol and NASH has prompted research into the use of cholesterol-lowering medications to protect subjects from developing NASH [60]. Hydrophobic Bas, such as deoxycholic acid, can trigger liver apoptosis and are increased in NASH by JNK1/p53/sirtuin 1 pathway [61]. Farnesoid X receptors (FXRs) are BA receptors that restrain the synthesis of BA so FXR agonists can protect from steatohepatitis and improve hyperlipidemia and insulin resistance in obese patients [62].

## 5. Molecular Mechanisms of Hepatocellular Lipid Metabolism

The incapacity of hepatocytes to dispose of excess FFAs results in lipoapoptosis, an essential feature of NASH. Apoptosis can occur in hepatocytes via intrinsic or extrinsic pathways [28]. The intrinsic mechanism is activated by intracellular stresses such as oxidative stress or by organelle dysfunction including ER stress and mitochondrial permeabilization, while the extrinsic pathway is initiated by the binding of death ligands, such as Fas or TNF-related apoptosis-inducing ligand (TRAIL), to their respective receptors [63]. Both intrinsic and extrinsic mechanisms converge on effector caspases to regulate apoptosis.

The role of oxidative stress in cell injury and death has not been established. Nevertheless, the absence of therapeutic efficacy data for antioxidants would argue against ROS as a major contributor to liver injury in NASH patients [64]. Some studies have demonstrated that excessive antioxidants can contribute to insulin resistance [65,66]. The induction of apoptosis seems to be crucial to the pathogenesis of lipotoxic injury in the liver and in other tissues, and activation of caspases and JNK may be major effectors of this process [67].

A constant excessive availability of saturated fatty acids (SFAs) likely generates lipid intermediates that will direct normal triacylglyceride formation toward induction of ER stress by accumulation of unfolded or misfolded proteins in the ER [68]. This perturbation promotes a state of oxidative stress that is conducive to a conserved adaptive response that activates signaling pathways, resulting in translational arrest, degradation of proteins, and production of antioxidants, to allow recovery and cell survival [69]. This adaptive response is activated by at least three ER stress sensors, including activating transcription factor 6 (ATF6), inositol requiring enzyme 1 (IRE1), and PKR-like ER kinase (PERK) [68]. Despite translational arrest of protein synthesis via eukaryotic initiation factor 2a, the failure to upregulate ATF4 and efficiently degrade proteins in response to activating X-box protein 1 may identify NASH patients at risk of progressing to cirrhosis because of incompetent degradation of unfolded proteins [70].

If the adaptive response machinery fails and the ER stress continues, the alert response is activated and promotes apoptosis [71]. IRE1, PERK, and ATF6 meet at one point with C/EBP homologous protein (CHOP), which forms a heteromeric complex with c-Jun to positively regulate p53-upregulated modulator of apoptosis expression, with subsequent activation of B-cell lymphoma 2-associated X (Bax) protein [72]. Accordingly mitochondrial membrane channel formation results in the release of cytochrome C into the cytosol, promoting activation of downstream effectors caspase-3 and 7, proteases that dismantle the cell and cause cell death by apoptosis. IRE1 can also bind tumor necrosis factor receptor-associated factor 2 (TRAF2) to activate apoptosis signal-regulating kinase 1 (ASK1) and downstream JNK to facilitate formation of the c-Jun/CHOP heteromeric complex. CHOP upregulates death receptors that sensitize hepatocytes to circulating death ligands [72]. Mitochondrial dysfunction may be increased by stress-induced release of calcium by the ER [70]. TLR4 is a pattern-recognition receptor that activates a pro-inflammatory signaling pathway in response to SFAs [73]. This pathway is initiated by recruiting adaptor molecules to Toll/IL-1 receptor domain containing adaptor protein and myeloid differentiation factor 88, which eventually leads to activation of nuclear factor κB with production of TNFα [72]. Binding of TNFα to the TNF receptor forms a complex consisting of TNF receptor associated death domain protein (TRADD), TRAF2, and receptor interacting protein (RIP). This complex activates a proapoptotic ASK1/JNK pathway that ultimately results in Bim activation and mitochondrial dysfunction [74]. The TRADD/TRAF2/RIP complex may be internalized, and after recruiting Fas-associated protein with death domain, the mitochondrial amplification loop is activated by caspase 8 [74]. The participation of lysosomes in apoptosis has also been described in patients with NASH. Upon cytosol-to-lysosome translocation of Bax and lysosomal membrane permeabilization, cathepsin B is released into the cytosol with subsequent increased production of TNFα through activation of the NF-κB pathway [75]. However, hepatocytes appear to be sensitized in patients with NASH and hepatic upregulation of the TRAIL receptor DR5 has been reported [76].

## 6. Lipids that Protect Against Lipotoxicity

Liver lipid metabolism is implicated in several diseases, including obesity, NAFLD, and T2DM [77]. It has been observed that hepatic accumulation of neutral lipids is a protective mechanism, while lipoperoxidation is implicated in the development and progression of NASH [78]. The gut microbiota (GM) has a very important role in liver lipid metabolism because it is involved in a complex modulation of metabolism [21]. Moreover, it has been observed that the translocation of the GM contributes to the maintenance of the low-grade inflammation present in MetS [79].

Recent studies have observed that the exposure of human and murine hepatocytes to monounsaturated fatty acids preserved hepatocyte viability despite lipid accumulation [80,81]. Therefore, monounsaturated FFAs are a safe form of excess lipid storage, and have been demonstrated to have a protective role against apoptosis in Huh-7 cells and primary hepatocytes [82]. In contrast, palmitoleic acid has not been shown to have a protective effect against steatosis, but can protect hepatocytes against the downstream death mediator Bax.

Two classes of long-chain polyunsaturated fatty acids (essential fatty acids) exist in nature: ω-3 and ω-6. They are structurally classified by the number of carbons, double bonds, and proximity of the first double bond to the methyl (omega) terminal of the fatty acid acyl chain [83]. Fatty acids of the ω-3 class have a double bond at the third carbon, whereas those of the ω-6 class have a double bond at the sixth carbon. ω-3 polyunsaturated fatty acids (PFAs) can limit triacylglycerol storage in the liver, while ω-6 PFAs can induce NAFLD [84].

Furthermore, it has been observed that ω-3 PFAs improve the human health by decreasing the visceral fat and decreasing the adipocyte size. This is because ω-3 PFAs are converted to anti-inflammatory eicosanoids, whereas ω-6 PFAs are converted to pro-inflammatory eicosanoids

In this connection, ω-3 PFAs have recently been suggested as a potential therapy for prevention and treatment of NASH [85]. A recent systematic review analyzed seven randomized control trials of ω-3 PFAs supplementation for NAFLD patients. The seven randomized control trials involved a total of 227 patients in the experimental groups and 215 in the control groups. This systematic review analyzed the values of alanine aminotransferase (ALT), aspartate aminotransferase (AST), γ-glutamyl transferase (GGT), total cholesterol (TC), TGs, low-density lipoprotein cholesterol (LDL-C), high-density lipoprotein cholesterol (HDL-C), fasting glucose, homeostatic model assessment insulin resistance (HOMA_IR_), adiponectin, liver fat, and fibrosis between both groups. The analysis showed that supplementation of ω-3 PFAs was efficient in patients with NAFLD in decreasing ALT and TC, mainly in reducing TGs, and increasing HDL-C. They also showed a tendency for ω-3 PFAs to reduce AST, GGT, and LDL-C. Nevertheless, ω-3 PFAs did not significantly improve liver fibrosis in patients with NAFLD. With regard to the dosage of ω-3 PFAs, the efficacy of ≥3 g treatment was better than <3 g, except for HDL-C. It seems that ω-3 PFAs in adults have liver therapeutic benefit but more studies are necessary to determine dose responsiveness, duration of therapy, safety, and patient tolerance before clinical recommendations can be made [86].

The availability of ω-3 long-chain PFAs to the liver is decisive for fat elimination from hepatocytes [87]. The beneficial effects of ω-3 PFAs were identified when significant decreases of ω-3 PFA, such as eicosapentaenoic and docosahexaenoic acids (fatty acids that may suppress progression of hepatic steatosis to NASH), were found in NASH patients [88,89].

## 7. Clinical Implications of Lipotoxicity

Elevated plasma levels of FFAs impair insulin signaling and produce skeletal muscle insulin resistance in healthy subjects [90]. Furthermore, the excess of FFAs leads to an increase in intramyocellular lipids [91].

**Figure 2:**
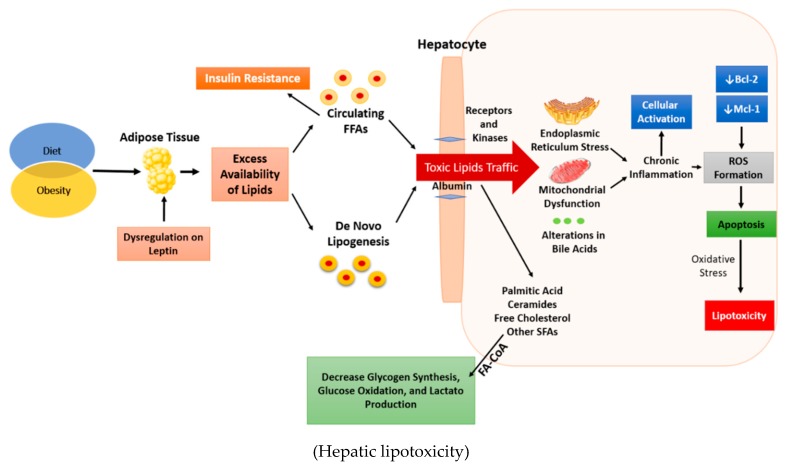
The increment in dietary energy availability and sedentary lifestyles leads to obesity. Obesity causes an excessive accumulation of lipids in the adipose tissue and in non-adipose tissue. Adipocytes provide a place to load and store energy in the form of triglycerides, protecting non-adipose tissues from immoderate accumulation of lipids because they have a restricted capacity to store triglycerides. This protection needs the permissive action of leptin, a hormone that decreases lipogenesis and increases oxidation in non-adipose tissue during acute overnutrition, dissipating the unnecessary energy of FFAs. The immoderate accumulation of FFAs exceeds the oxidative capacity of non-adipose tissues, thus potentiating the metabolic flux of fatty to other noxious non-oxidative pathways producing specially ceramides. The increment in FFAs increases tumor necrosis factor (TNF)-α, which promotes insulin resistance, and ROS, which trigger hepatocyte apoptosis. Also, SFAs induce endoplasmic reticulum stress, which leads to the upregulation of the pro-apoptotic BH3-only proteins (BIM and PUMA), resulting in inactivation of the antiapoptotic Bcl-2 family members (Mcl-1 and Bcl-xL). SFAs: saturated fatty acids, ROS: reactive oxygen species, FA-CoA: Fatty-acyl-CoA synthase, FFAs: free fatty acids.

It has been observed that skeletal muscle insulin resistance is closely related to a diversity of lipid-derived toxic metabolites from incomplete oxidation of FFAs, including acylcarnitines and long-chain fatty acyl CoAs, DAG, and/or ceramides [27] (Figure 2).

The clinical implication of these data is that even a mild increment in FFA levels in AT and plasma may produce muscle lipotoxicity. Consequently, it is not surprising that obese patients with NAFLD as well as non-obese individuals with NASH combined with AT insulin resistance have muscle insulin resistance [92]. The metabolic behavior of obese individuals who do not have AT insulin resistance is similar to that of lean individuals, with near-normal muscle insulin sensitivity; hence this group of obese individuals usually does not develop NAFLD [93].

Several studies have reported that increased plasma FFA levels are related closely to T2DM [94,95]. Although the underlying mechanisms are poorly understood, diabetes is associated with more severe liver disease. It has been observed that T2DM is associated with a two- to four-fold increase in serious liver disease, such as cirrhosis and hepatocellular carcinoma [96,97]. In this connection, T2DM in NAFLD patients is associated with more severe hepatic and AT insulin resistance as well as higher values of NAFLD activity score, and thus advanced liver fibrosis [98,99]. It is important to mention that the high levels of FFAs not only induce hepatic insulin resistance but also impair insulin clearance, leading to hyperinsulinemia in insulin-resistant states and in patients with NAFLD [100].

Many studies have reported a strong correlation between NAFLD and a high risk of developing cardiovascular disease (CVD). This association is currently not well understood [100,101,102,103,104]. It is well-known that NAFLD patients have abnormal endothelial function [105]. Several studies have reported that subjects with increased plasma aminotransferase concentrations have a higher Framingham risk score and/or number of cardiovascular events compared with those with normal levels [106,107,108]. The current data may not be reliable because study designs led to substantive biases including incomplete diagnosis of NAFLD, inadequate adjustment for confounders, and only short-term follow-up. Thus, long-term prospective studies are necessary to establish and confirm this relationship.

## 8. Treatment of Lipotoxic Liver Injury

### 8.1. Lifestyle Modification

Lifestyle interventions can result in weight loss, decrease CVD, improve fibrosis, and delay the progress of T2DM [109,110,111,112]. Weight loss enhances the levels of aminotransferases and hepatic steatosis, as measured either by ultrasonography or magnetic resonance spectroscopy in proportion to the total quantity of weight lost [113]. It has been reported that NASH patients require a weight reduction of >10% to induce near-universal resolution of NASH or to reduce fibrosis by at least one stage [114].

Regular physical activity of moderate intensity for at least six to 12 weeks without dietary modification has been shown to decrease hepatic steatosis in NAFLD patients [115]. Moreover, lifestyle modification therapy has beneficial effects in treating MetS because it reduces the risk of coronary heart disease and the development of T2DM in patients with NAFLD [116].

### 8.2. Pharmacologic Interventions in NASH

There are no currently approved pharmacologic therapies for NASH, but vitamin E and pioglitazone hold promise for the treatment of NASH patients [117].

#### 8.2.1. Vitamin E

The mechanism of action of vitamin E is probably related to amelioration of intracellular oxidative stress in NASH patients [118]. Some studies have demonstrated that vitamin E 800 IU/day improves liver histology in NASH patients, including reduction of liver steatosis and lobular inflammation [119,120]. Although the use of vitamin E (800 IU/day) is currently recommended only for patients with biopsy-proven NASH and without diabetes, some studies have shown histological improvement with vitamin E treatment regardless of diabetes status [121,122,123]. Nowadays, vitamin E has demonstrated safety at dosages of less than 1500 IU/day [124,125]. Nevertheless, long-term or high-dose vitamin E treatment may be related to an increase in all-cause mortality, prostate cancer, and hemorrhagic stroke [126,127,128].

#### 8.2.2. Pioglitazone

Pioglitazone is a drug of the thiazolidinedione (TZD) class. The main molecular target of TZDs is the PPAR-γ in AT [129,130]. This translates into several metabolic and anti-inflammatory effects.

Pioglitazone treatment has been shown to lead to improvement in advanced fibrosis and in fibrosis of any stage in NASH patients, even in those without diabetes. This drug can reduce subclinical inflammation, improve AT and hepatic insulin signaling, and enhance insulin action [131,132]. Moreover, pioglitazone improves the metabolic profile of non-diabetic patients with NAFLD, and may reduce the risk of progression from prediabetes to T2DM [133]. Several studies suggest that pioglitazone confers protection against cardiovascular events because it has been observed that this drug improves dyslipidemia and reduces vascular inflammation. Unfortunately, pioglitazone has been shown to have secondary effects related to weight gain [133,134].

### 8.3. Bariatric Surgery

Several studies have suggested that bariatric surgery reduces weight, insulin resistance, alterations in glucose metabolism, hypertension, transaminase levels, and histological changes related to simple steatosis, NASH, and fibrosis [135]. Bariatric surgery is not currently recommended as a first-line treatment for NAFLD and NASH. Those patients with a BMI >40 kg/m^2^ or with BMI >35 kg/m^2^ and associated comorbidities can benefit from bariatric surgery because this procedure has been shown to lead to improvements in metabolism and reduce cardiovascular and general mortality [136]. There are studies reporting the beneficial effects of bariatric surgery in subjects with a lower BMI in the presence of associated comorbidities, specifically T2DM [137]. Unfortunately, the safety and efficacy of bariatric surgery in eligible obese individuals with NAFLD-related cirrhosis have not been established.

#### Endoscopic Weight Loss

Endoscopic options for induction of weight loss are becoming very popular, which is why there is a growing literature of endoscopic procedures for weight loss in the treatment of NAFLD patients [138]. The most studied endoscopic procedure is the endoscopic gastric balloon, a restrictive endoscopic modality [139]. This endoscopic technique has shown a significant effect on parameters of NAFLD, such as AST and ALT. In this connection, Lee et al. have performed a prospective study to evaluate the effects of an intragastric balloon on the NASH histology in obese patients [140]. They observed a significant improvement in liver steatosis and NAFLD activity score after six months in the treatment group. Nevertheless, their results are inconclusive due to their sample being small and the follow-up time very short. Further studies are needed to establish the long-term safety and efficacy of endoscopic weight loss techniques in the treatment of NAFLD.

### 8.4. Dietary Supplements

#### 8.4.1. Milk Thistle

*Silybum marianum* or milk thistle (MT) is a member of the Asteraceae/Compositae family. It has been used in the treatment of liver diseases for millennia due to its anti-inflammatory properties that may be beneficial in NAFLD [141]. The active complex of MT is composed of isomer flavonolignans (silibinin, isosilibin, silidianin, and silichristine), collectively known as silymarin. Silibinin is the main isomer and the most active component and represents about 60–70% of MT [141]. Silymarin can decrease lipid peroxidation and free radical production. Moreover, Silymarin can induce the apoptosis of hepatic stellate cells or the degradation of collagen deposits [142,143]. Nevertheless, silibinin has a low bioavailability but it can be enhanced by complexation with phosphatidylcholine or β-cyclodextrin. Several studies have demonstrated that silymarin and silibinin have hepatoprotective properties such as action on insulin resistance, lipid peroxidation, and restoration of glutathione levels that lead to improvements in liver esteatosis and fibrosis regression [144,145,146,147,148,149]. Unfortunately, the standardization of silymarin in its several formulations and effective doses is still lacking.

#### 8.4.2. Coffee

The consumption of coffee is related to a decreased risk of cirrhosis, hepatocellular carcinoma, and mortality [150]. Several studies have observed that regular coffee intake is significantly associated with reduced hepatic fibrosis of NAFLD [151,152]. Currently studies have suggested a beneficial effect of coffee on the liver to reduce liver enzymes [153,154,155]. Although there is growing research in this area, the exact role caffeine plays in NAFLD progression remains unknown and there is no clear consensus on what amount of coffee confers the greatest decreased risk of liver fibrosis [151]. Finally, animal studies showed the importance of coffee, independently of the caffeine content, in preventing the progression of NASH [156]. In this study, it was observed that NAFLD rats that were given decaffeinated coffee had lower levels of liver fat and collagen, decreased liver oxidative stress through glutathione metabolism, and lower liver inflammation compared to control rats that did not consume coffee.

#### 8.4.3. Gut Microbiota

GM is implicated in obesity and the development of MetS. It has been observed that disturbed gut-liver barrier integrity contributes in the pathogenesis of NAFLD and NASH because bacteria and their products can escape into the circulation, producing a massive inflammatory response [22]. To date, there are some drugs that have an effect on GM.

IMM-124e is an IgG-enhanced bovine-derived colostrum that has been developed to target LPS in the gut in order to prevent the translocation of bacteria and their products (LPS) into the portal circulation [157]. Although this is an experimental drug, it has demonstrated significant reduction of serum LPS levels.

Orlistat is an FDA-approved lipase inhibitor to treat obesity. This drug has a direct effect in the gut to reduce dietary fat absorption. Orlistat seems to improve liver enzyme levels and liver content, but its effectiveness in NASH has not yet been evaluated [158].

## 9. Emerging Therapies

In recent years, new drugs for NASH treatment have been created [159]. These substances include peroxisome proliferator-activated receptors agonists (Elafibranor), FXR agonists (obethicolic acid), dual FXR/G protein-coupled receptor 5 agonists (6α-ethyl-3α,7α-dihydroxy-24-nor-5β-cholan-23-sulfate: INT-767) and bile acids sequestrants (colesevelam and cholestyramine) [160]. Currently, the most promising new drugs are FXR agonists that have shown an increase in insulin sensitivity and decreased markers of hepatic inflammation and fibrosis in NAFLD patients with T2D [161].

## 10. Conclusions

Lipotoxicity implicates very complex cellular mechanisms in which the hepatic accumulation of FFA results in hepatocyte dysfunction or hepatocyte cell death. However, the pathogenesis of liver fibrosis progression in NASH is not yet completely understood. Therefore, knowledge of the molecular mechanisms behind hepatocellular injury is crucial to reduce or avoid liver fibrosis. Further studies are necessary to understand the crosstalk between the liver and AT on a molecular level and how it affects the progression from liver fibrosis to cirrhosis and to hepatocellular carcinoma. Future therapeutic concepts for NAFLD and NASH will have to take into account novel concepts of lipotoxic liver injury.

## Abbreviations

ASK1	Apoptosis signal-regulating kinase 1
ALT	Alanine aminotransferase
AST	Aspartate aminotransferase
AT	Adipose tissue
ATF	Activating transcription factor
BA	Bile acid
BAT	Brown adipose tissue
Bax	B-cell lymphoma associated X
CCAAT	Cytosine–cytosine–adenosine–adenosine–thymidine
CHOP	C/EBP homologous protein
CoA	Coenzyme A
CVD	Cardiovascular disease
DAG	Diacylglycerol
ER	Endoplasmic reticulum
FC	Free cholesterol
FFAs	Free fatty acid
FXR	Farnesoid X receptor
GM	Gut microbiota
IL	Interleukin
IRE1	Inositol requiring enzyme 1
JNK	Jun terminal kinase
LPC	lysophosphatidyl choline
MT	Milk thistle
MetS	Metabolic syndrome
NAFLD	Nonalcoholic fatty liver disease
NASH	Nonalcoholic steatohepatitis
N-SMase	Neutral sphingomyelinase
OA	Oleic acid
PA	Palmitic acid
PFA	Polyunsaturated fatty acid
PERK	PKR-like ER kinase
PPAR-γ	Peroxisome proliferator-activated receptor gamma
RIP	receptor interacting protein
ROS	Reactive oxygen species
SFA	Saturated fatty acid
SREBP	Sterol regulatory-element binding protein
T2DM	Type 2 diabetes mellitus
TC	Total cholesterol
TGs	Triglycerides
TLR4	Toll-like receptor 4
TNFα	Tumor necrosis factor alpha
TRADD	TNF receptor associated death domain protein
TRAF2	Tumor necrosis factor receptor-associated factor 2
TRAIL	TNF-related apoptosis-inducing ligand
WAT	White adipose tissue
